# Non-energy devices to dissect recurrent laryngeal nerve lymph nodes of non-small cell lung cancer under video-assisted thoracic surgery

**DOI:** 10.1186/s12893-021-01179-2

**Published:** 2021-03-30

**Authors:** Minhao Yu, Mingjian Ge

**Affiliations:** 1 Department of Thoracic Surgery, Chengdu BOE Hospital, Chengdu, 610200 China; 2 Department of Thoracic Surgery, The First Affiliated Hospital of Chongqing Medical University, No.1 Youyi Road, Yuzhong District, Chongqing, 400016 China

**Keywords:** Non-small cell lung cancer, Recurrent laryngeal nerve injury, Non-energy device, Systematic nodal dissection

## Abstract

**Background:**

Systematic nodal dissection plays a crucial role in improving survival and staging in resectable non-small cell lung cancer (NSCLC) patients but at the cost of increasing the occurrence of recurrent laryngeal nerve injury. Technology should be improved to protect the recurrent laryngeal nerve (RLN) during surgery.

**Methods:**

NSCLC patients who underwent video-assisted thoracic surgery (VATS) surgical treatment by the same surgeon at our hospital from January 2016 to December 2017 were included as the research subjects and were divided into an energy-device group and a non-energy-device group. Their procedures included anatomic pulmonary resection, normative N1 dissection, and systemic N2 dissection.

**Results:**

The rate of metastatically involved recurrent laryngeal nerve lymph nodes (RLNLNs) was 5.19% (39/752). Dissection device, side of primary, FEV1, operative time and BMI were independent predictors of recurrent laryngeal nerve injury (RLNI) (hazard ratio (HR) = 3.576, 95% confidence interval (CI): 1.490–8.583, P = 0.004; HR = 0.175, 95% CI: 0.072–0.424, P =  < 0.001; HR = 3.008, 95% CI: 1.30–6.927, P = 0.010; HR = 0.328, 95% CI: 0.136–0.794, P = 0.013; HR = 0.344, 95%CI: 0.147–0.801, P = 0.013, respectively). Patients in the non-energy-device group had significantly less RLNI than the energy-device group (P = 0.016) and nearly half of the non-thermal RLNI recovered in 2 weeks (P = 0.025) whereas most thermal RLNI required 3 months for recovery.

**Conclusions:**

Every station of RLNLN had some degree of cancer metastasis in NSCLC patients and when dissecting RLNLNs, dissection device was an independent and artificially controlled predictor of RLNI. Using a non-energy device is a feasible method to protect the RLN as well as an improved recovery time of RLNI.

## Background

Lung cancer was the most common incident cancer and the leading cause of cancer death in China in 2015, with a total of 733,300 new cases and 610,200 deaths [1]. For patients with resectable non-small cell lung cancer (NSCLC), complete resection should be performed, and to achieve a radical resection, systematic nodal dissection (SND) is essential. A study from Wu et al. concluded lobectomy combined with SND can improve survival in resectable NSCLC, especially for patients in Stage I and Stage IIIA [2]. Although some researchers have reported that lobe-specific lymph node dissection has the potential to be a standard procedure in the surgical treatment of NSCLC [3], Maniwa et al. [4] found that recurrence of metastatically involved mediastinal nodes in patients undergoing lobe-specific nodal dissection was significantly greater than those undergoing SND. Wen et al. [5] also provided evidence that at least 12 locations of lymph nodes should be examined to provide longer 5-year cancer specific survival and a significant reduction in disease recurrence in patients with T2N0 NSCLC. Additionally, SND is also important for disease staging. Alastair et al. [6] postulated that because no clinical or pathologic subset of patients could be identified that had a negligible incidence of N2 disease, SND should be routinely undertaken for accurate intrathoracic staging of NSCLC. Even though there are alternative sampling technologies, such as EBUS-TBNA and mediastinoscopy, a Dutch study showed that increased usage of less invasive endosonography prior to or even substituting for surgical staging did not uncover unexpected cases of N2 nodal disease in NSCLC [7], therefore, the greater the number of resected lymph nodes, the less likely N1 or N2 disease would be incorrectly diagnosed as N0 [5]. Furthermore, in Liu`s study, the incidences of upstaging from N0 to N1 and N2 disease were 7.7% and 12.2% utilizing SND [8].

Nevertheless, the role of SND in the treatment of NSCLC remains controversial, because a more thorough lymph node dissection typically increases the occurrence of postoperative complications. These include bleeding, cardiac arrhythmias, chylothorax, bronchopleural fistula and recurrent laryngeal nerve injury (RLNI). When dissecting the recurrent laryngeal nerve lymph nodes (RLNLNs), defined as 2R, 3p, 4L and 5 lymph nodes, the recurrent laryngeal nerve (RLN) may be damaged with significant neurological consequences. A study from Ewald et al. [9] verified that all unintentional injuries of the RLN were associated with mediastinal lymph node dissection (MLND). Watanabe et al. [10] reviewed their technique and experience of MLND, and found their incidence of RLNI to be 1.5%. Notably, they performed all MLND using thermal technology including conventional electrocautery or ultrasonic scalpel. Experimentally, a decrease in RLNI was reported with the implementation of the vessel sealing system (VSS) when compared with conventional scissors [10]. But no further investigation evaluated the risk factors for RLNI.

Because it was our impression from our own experience, that RLNI was infrequent using traditional non-energy devices, we tested this hypothesis by retrospectively examining the frequency of RLNI following RLNLN dissection using either “energy” and “non-energy” devices, during NSCLC surgery.

## Materials and methods

### Patients

Included were selected NSCLC patients who underwent surgical treatment by the same surgeon at The First Affiliated Hospital of Chongqing Medical University, China from January 2016 to December 2017, divided into an energy-device group and a non-energy-device group. In addition to this, we confirm that all methods were performed in accordance with the relevant guidelines and regulations. The inclusion criteria were as follows: NSCLC patients with clinically early (cT1a-2bN0-1M0) and advanced (cT3-4N0-1MO) stage disease, without invasion of surrounding tissues, who underwent surgical treatment via video-assisted thoracic surgery (VATS). Exclusion criteria were as follows: preoperative hoarseness or choking, neoadjuvant therapy, intraoperative RLNLN calcification and fusion, intraoperative tumor or metastatic lymph node invasion of the RLN; intrathoracic extensive adhesions or pleural cavity atresia, non-anatomical lung resection, and segmentectomy. The final study group comprised 188 patients.

### Surgical methods

Using a VATS approach, all patients underwent anatomic pulmonary resection, normative N1 dissection, and systemic N2 dissection. The dissection zones of the right N2 lymph node included 2R, 3a, 3p, 4R, 7, 8R, and 9R, whereas the left dissection zones included 4L, 5, 6, 7, 8L, and 9L. The energy-device group underwent RLNLNs dissection using energy devices such as an electric hook or electrotome. In the non-energy-device group, devices such as tissue scissors or endoscopic scissors were used. In both groups, the initial dissection exposed the motor and sensory branches of the RLN, and the motor branches were carefully protected. The sensory branches were transected to expose the deep lymph node mass. During dissection, the RLN was kept in place to avoid mechanical stretch.

### Determination of RLNI

The occurrence of RLNI was determined by the clinical presence of postoperative hoarseness and/or choking.

### Statistical analysis

SPSS 22.0 (IBM Corp., Armonk, NY, USA) software was used for the statistical analysis. Continuous data were expressed as the mean ± standard deviation. A t-test was used to compare normally distributed data according to whether the variance was equal or not. The Mann–Whitney U test was used to compare non-normally distributed data sets. Categorical data were expressed as counts and percentages, and comparisons between groups were performed using the chi-square test or the Fisher exact probability method. Indicators with statistically significant results from univariable analysis were incorporated into a logistic regression model for multivariable analysis to screen for the risk factors of RLN injury. We used Propensity Scores Matching (PSM) to adjust selection bias. The difference was statistically significant at P < 0.05.

## Results

### Patients characteristics

Of the 188 NSCLC patients, there were 119 men and 69 women. The age, body mass index (BMI), forced expiratory volume in 1 s (FEV1), location of primary, lobar origin, stage, mean tumor diameter and blood loss between the two groups were not significantly different, whereas the operative time in the non-energy-device group was significantly lower than the energy-device group (P = 0.006). The clinical characteristics of the two groups are shown in Table 1.

**Table 1:** Comparison of clinical characteristics between the two groups

Clinical characteristics	Groups		P value
	Non-energy device (n=96)	Energy device (n=92)	
Age (years)	60.55±9.82	60.84±9.32	0.839
Gender			
Male	58 (30.85%)	61 (32.45%)	0.450
Female	38 (20.21%)	31 (16.49%)	
BMI	22.92±3.12	23.39±3.57	0.347
FEV1	2.36±0.58	2.27±0.66	0.384
Location of primary			
Peripheral	80 (42.55%)	69 (36.70%)	0.208
Central	16 (8.51%)	23 (12.23%)	
Lobar origin			
RUL	19 (10.11%)	32 (17.02%)	0.277
RML	12 (6.38%)	5 (2.66%)	
RLL	16 (8.51%)	14 (7.45%)	
RMLL	0 (0%)	2 (1.06%)	
Right hilum	1 (0.53%)	2 (1.06%)	
LUL	33 (17.55%)	18 (9.57%)	
LLL	15 (7.98%)	16 (8.51%)	
Left hilum	0 (0%)	3 (1.60%)	
pTNM stage			
IA	35 (18.62%)	17 (9.04%)	0.084
IB	19 (10.11%)	26 (13.83%)	
IIA	5 (2.66%)	6 (3.19%)	
IIB	14 (7.45%)	13 (6.91%)	
IIIA	15 (7.98%)	19 (10.11%)	
IIIB	2 (1.06%)	9 (4.79%)	
IIIC	1 (0.53%)	0 (0%)	
IVA	4 (2.13%)	3 (1.60%)	
Mean tumor diameter (cm)	3.21±1.92	3.30±1.45	0.736
Operative time (min)	154.98±43.07	172.83±44.96	0.006
Blood loss (ml)	216.76±148.06	188.37±105.70	0.133
Chest tube drainage (ml)	117.62±51.34	104.86±50.29	0.352
Duration with tube (day)	5.49±3.82	3.97±3.27	0.531
RLNI	12 (6.38%)	25 (13.30%)	0.016

*BMI* body mass index, *FEV1* forced expiratory volume in 1 second, *RUL* right upper lobe, *RML* right middle lobe, *RLL* right lower lobe, *RUML*right upper-middle lobe, *RMLL* right middle-lower lobe, *LUL* left upper lobe, *LLL* left lower lobe, *RLNI* recurrent laryngeal nerve injury

### Rate of metastatically involved RLNLNs

Four RLNLN stations were dissected in all 188 patients for a total of 752 stations. Pathologically confirmed metastatic RLNLNs were found in 39 stations form 37 patients, yielding a metastatic rate of 5.19% (39/752, Table 2).

**Table 2:** Metastatic rate of RLNLNs

Station	n	Metastatic rate
2R	15	1.99% (15/752)
3p	3	0.40% (3/752)
4L	10	1.33% (10/752)
5	11	1.46% (11/752)
Total	39	5.19% (39/752)

*RLNLNs* recurrent laryngeal nerve lymph nodes

### Risk factors of RLNI

Univariable analysis using PSM to preclude bias, identified dissection device, side of primary, FEV1, operative time and BMI as significantly associated with RLNI; age, gender, stage and location of the primary tumor were not similarly associated (Table 3). The significantly associated variables were then assessed by logistic regression model analysis, and all proved to be independent predictors of RLNI (Table 4).

**Table 3:** Univariable analysis of RLNI pre- and post-PSM

	RLNI (pre-PSM)			RLNI (post-PSM)		
	Yes (n=37)	No (n=151)	P value	Yes (n=24)	No (n=24)	P value
Dissection device						
Energy	25 (13.30%)	67 (35.64%)	0.016	11 (22.92%)	3 (6.28%)	0.008
Non-energy	12 (6.38%)	84 (44.69%)		13 (27.08%)	21 (43.75%)	
Side of primary						
Left	26 (13.83%)	59 (31.38%)	0.001	8 (16.67%)	8 (16.67%)	< 0.001
Right	11 (5.85%)	92 (48.94%)		16 (33.33%)	16 (33.33%)	
Age (years)						
< 60	20 (10.64%)	61 (32.45%)	0.143	12 (25.00%)	16 (33.33%)	0.506
> 60	17 (9.04%)	90 (47.87%)		12 (25.00%)	8 (16.67%)	
Gender						
Male	22 (11.70%)	97 (51.60%)	0.704	16 (33.33%)	15 (31.25%)	0.806
Female	15 (7.98%)	54 (28.72%)		8 (16.67%)	9 (18.75)	
FEV1 (L)						
< 2	23 (12.23%)	59 (31.38%)	0.016	13 (27.08%)	15 (31.25%)	0.012
≥ 2	14 (7.45%)	92 (48.94%)		11 (22.92%)	9 (18.75)	
BMI						
< 24	18 (9.57%)	96 (51.06%)	0.132	11 (22.92%)	11 (22.92%)	0.011
≥ 24	19 (10.11%)	55 (29.26%)		13 (27.08%)	13 (27.08%)	
Operative time (min)						
< 150	11 (5.85%)	74 (39.36%)	0.043	16 (33.33%)	12 (25.00%)	0.022
≥ 150	26 (13.83%)	77 (40.96%)		8 (16.67%)	12 (25.00%)	
Location of primary						
Peripheral	28 (14.89%)	121 (64.36%)	0.651	18 (37.50%)	16 (33.33%)	0.901
Central	9 (4.79%)	30 (15.96%)		6 (12.50%)	8 (16.67%)	
Stage						
Early	21 (11.17%)	113 (60.11%)	0.042	8 (16.67%)	6 (12.50%)	0.056
Advanced	16 (8.51%)	38 (20.21%)		16 (33.33%)	18 (37.50%)	

*BMI* body mass index, *FEV1* forced expiratory volume in 1 second, *RLNI* recurrent laryngeal nerve injury, *PSM* propensity scores matching

**Table 4.** Multivariable analysis of RLNI

	HR (95% CI)	P value
Dissection device (energy vs. non-energy)	3.576 (1.490–8.583)	0.004
Side of primary (left vs. right)	0.175 (0.072–0.424)	< 0.001
BMI (< 4 vs. ≥24)	0.344 (0.147–0.801)	0.013
FEV1 (< 2 vs. ≥2)	3.008 (1.307–6.927)	0.010
Operative time (< 150min vs. ≥150min)	0.328 (0.136–0.794)	0.013

*BMI* body mass index, *HR* hazard ratio, *CI* confidence interval, *FEV1* forced expiratory volume in 1 second, *RLNI* recurrent laryngeal nerve injury

### Recovery time of RLNI

If either hoarseness or choking persisted, the neurological function of RLNI was considered not recovered. On the contrary, if both resolved, this completely recovered. With ongoing follow-up, nearly half of the RLNI patients in the non-energy-device group had recovered by two weeks. In contrast, only two patients had recovered in the energy-device group (P = 0.025). There was one patient in the non-energy-device group and five in the energy-device group that had not recovered by six months postoperatively, but the difference was not significant (P = 0.641, Table 5).

**Table 5:** Comparison of the recovery time of RLNI between the two groups

	Non-energy device (n=12)	Energy device (n=25)	P value
2 weeks	5 (41.67%)	2 (8.00%)	0.025
1 month	3 (25.00%)	7 (28.00%)	0.998
3 months	3 (25.00%)	11 (44.00%)	0.306
More than 6 months	1 (8.33%)	5 (20.00%)	0.641

*RLNI* recurrent laryngeal nerve injury

## Discussion

Mediastinal lymph node staging is an important component of the assessment and management of patients with operable NSCLC and is necessary to achieve complete resection [10]. In our study, every station of RLNLN had some degree of cancer metastasis and postoperative examination confirmed that the metastatic rate of RLNLNs was 5.2% (Table 2) for unforeseen N2 NSCLC patients. Although a relatively low percentage, the presence of metastatic RLNLNs had important implications in guiding postoperative treatment, controlling local recurrence rates, and judging patient prognosis. A 10-year cohort study showed patients that underwent MLND had longer survival than those with only mediastinal lymph node sampling (154.67 months vs. 124.67 months) [11]. Many thoracic surgeons are reluctant to proceed with dissection of RLNLNs due to its particular anatomical structure and the voice and swallowing changes incurred by its damage [12]. The RLN gives off sensory and motor branches along its course to the larynx, and these branches are woven into webs at the RLN looping point and surround the lymph nodes and adipose tissue [13, 14].

The risk factors of RLNI are varied. In our study, having excluded factors that might have influenced the technical aspects of the operation, such as neoadjuvant therapy, intraoperative RLNLNs calcification and fusion, intraoperative tumor or metastatic lymph node invasion of the RLN and intrathoracic extensive adhesions or pleural cavity atresia, and precluded bias using PSM, we found that the dissection device, side of primary tumor, FEV1, operative time and BMI were significantly associated with RLNI (Table 3), and all of them were independent predictors of RLNI (Table 4). All of these factors were out of control by the surgeon except the specific type of dissection device. Therefore, improvement in dissection technique, and in this study, dissection technology, could facilitate attaining the goal of reducing RLNI.

In the present study, patients in the non-energy-device group had significantly fewer RLNI than the energy-device group (Table 1). This was consistent with previous research indicating that the RLN could be paralyzed by thermal injury [10]. The results seem clear, but the procedure is difficult, because the anatomic detail around the RLNLNs is complex. The key to safe dissection of the RLNLNs is to identify and protect the motor branches of the RLN, which are the principal supply to the vocal cords [15]. Fortunately, the RLN’s motor branches are larger in diameter with a constant course. These can be distinguished by the naked eye; the RLN function will not be affected when the sensory branches are separated [16]. However, some surgeons may be worried about chylothorax necessitating long duration of a chest tube, thinking that the routine use of the VSS can reduce postoperative chest tube drainage and earlier removal of the chest tube after MLND [10]. However, the present study showed that the duration of postoperative drainage in the non-energy-device group was not significantly longer than the energy-device group as long as the small vessels were carefully identified and clipped (Johnson, ER320).

All types of injury to the motor branches of the RLN can cause laryngeal muscle dysfunction, irrespective of thermal or non-thermal injury. Animal experiments showed that the RLN stimulation signal was not changed following continuous stimulation of the RLN at 40–55 °C for 60 s, but the signals were not received with RLN stimulation applied at 60 °C for 30 s, or 70 °C for 20 s[17]. This study also showed that when the temperature exceeded 60 °C, the RLN had been damaged [17]. Importantly, therefore, the temperature measured by Applewhite when the ultrasonic dissector was activated was approximately 115 °C, and the temperature generated by unipolar or bipolar coagulation was also approximately 100 °C [18]. Therefore, if the energy device touched the RLN during cutting or hemostasis, it would likely cause permanent RLNI for it always damaged the endoneurium [19]. Non-energy devices can also cause RLNI when scissors conduct vessels which reach the lymph nodes, and that may cause local hemorrhage due to non-vascular occlusive effects, resulting in stretch or clot compression when hemostasis. Schneider et al. [20] observed real-time RLN monitoring of animal experiments and found that when the RLN was stretched with a tensile force of 3000 mN, the signal received by the electromyography weakened by approximately 59%; when the RLN was compressed by 280 mmHg (37 KPa), the signal received by electromyography weakened by about 40%. However, most signals could recover again after releasing the traction or compression. Therefore, non-thermal RLNI is usually temporary.

Although it is very difficult to accurately assess RLNI without laryngoscopy [21], we relied on the clinically useful symptoms of hoarseness and choke as indicators of RLNI. Hoarseness was a common clinical manifestation of RLNI, and 79% of RLN with surgical trauma had hoarseness [22]. In Ewald 's study, he also equated hoarseness with RLNI [9]. Choke was mostly manifested as impairment of the superior laryngeal nerve (SLN) internal branch [23], but some patients with RLNI could also manifest as choke. The mechanism for this is the ipsilateral vocal cord loses its motor function and is fixed in the median or paramedian position after RLNI, and the vocal cord loses its barrier function. Additionally, both the RLN and the SLN originate from vagus nerve (VN) [24]. Before the external branch of the SLN enters the cricothyroid muscle, it divides a branch together with the RLN to control the thyroarytenoid muscle and lateral cricoarytenoid muscle [25]. There may be a traffic branch between RLN and SLN [26]. There is also direct communication between the posterior branches of the RLN and the internal branch of the SLN [27], that provides sensory innervation to the laryngeal mucosa [23]. Therefore, the two indicators were reasonable. Real-time RLN intraoperative monitoring may be an alternative, but it is not widely available to district hospitals and is difficult to perform routinely in clinical practice.

We also compared recovery time of RLNI between the two groups and found nearly half of the non-thermal RLNI had recovered within two weeks, while most thermal RLNI required three months for recovery (Table 5). This indicated that if RLNI had occurred, neurological function would recover sooner when using non-energy devices. There was more RLNI that did not recover by 6 months in the energy-device group, but this was not statistically significant, perhaps due to the relatively small overall number or short duration of follow-up.

The operative time in the energy-device group was significantly longer than the non-energy-device group. The important reason for this was that when energy devices were used to dissect lymph nodes and its surrounding tissues that contain the sensory branches of the RLN, these need to be carefully identified and transected without using energy, as thermal injury could be conducted to the motor branches. However, when using non-energy devices, after careful identification of the motor branches, the tissue bundles could be sealed directly with clips, and then be severed simultaneously, without causing RLN function damage.

This is a retrospective study and the determination of RLNI was dependent on clinical assessment of hoarseness and choking. A more precise technique using real-time RLN intraoperative monitoring would be optimal for future prospective research.

## Conclusion

Every station of RLNLN had some degree of cancer metastasis in NSCLC patients and when dissecting RLNLNs, dissection device was an independent and artificially controlled predictor of RLNI. Using a non-energy device is a feasible method to protect the RLN as well as an improved recovery time of RLNI.

## Data Availability

All data generated or analysed during this study are included in this published article.

## Abbreviations

NSCLC: Non-small cell lung cancer
SND: Systematic nodal dissection
RLN: Recurrent laryngeal nerve
RLNLNs: Recurrent laryngeal nerve lymph nodes
RLNI: Recurrent laryngeal nerve injury
HR: Hazard ratio
CI: Confidence interval
MLND: Mediastinal lymph node dissection
VSS: Vessel sealing system
VATS: Video-assisted thoracic surgery
BMI: Body mass index
FEV1: Forced expiratory volume in 1 s
SLN: Superior laryngeal nerve
RUL: Right upper lobe
RML: Right middle lobe
RLL: Right lower lobe
RUML: Right upper-middle lobe
RMLL: Right middle-lower lobe
LUL: Left upper lobe
LLL: Left lower lobe
PSM: Propensity scores matching
